# Microbiomes of commercially-available pine nuts and sesame seeds

**DOI:** 10.1371/journal.pone.0252605

**Published:** 2021-06-21

**Authors:** Megan Fay, Joelle K. Salazar, Padmini Ramachandran, Diana Stewart

**Affiliations:** 1 Division of Food Processing Science and Technology, U. S. Food and Drug Administration, Bedford Park, Illinois, United States of America; 2 Division of Microbiology, U. S. Food and Drug Administration, College Park, Maryland, United States of America; University of Salento, ITALY

## Abstract

Metagenomic analysis of food is becoming more routine and can provide important information pertaining to the shelf life potential and the safety of these products. However, less information is available on the microbiomes associated with low water activity foods. Pine nuts and sesame seeds, and food products which contain these ingredients, have been associated with recalls due to contamination with bacterial foodborne pathogens. The objective of this study was to identify the microbial community of pine nuts and sesame seeds using targeted 16S rRNA sequencing technology. Ten different brands of each seed type were assessed, and core microbiomes were determined. A total of 21 and 16 unique taxa with proportional abundances >1% in at least one brand were identified in the pine nuts and sesame seeds, respectively. Members of the core pine nut microbiome included the genera *Alishewanella*, *Aminivibrio*, *Mycoplasma*, *Streptococcus*, and unassigned OTUs in the families of *Desulfobacteraceae* and *Xanthomonadaceae*. For sesame seeds, the core microbiome included *Aminivibrio*, *Chryseolina*, *Okibacterium*, and unassigned OTUs in the family *Flavobacteriaceae*. The microbiomes of these seeds revealed that these products are dominated by environmental bacterial genera commonly isolated from soil, water, and plants; bacterial genera containing species known as commensal organisms were also identified. Understanding these microbiomes can aid in the risk assessment of these products by identifying food spoilage potential and community members which may co-enrich with foodborne bacterial pathogens.

## Introduction

Next generation sequencing, including high-throughput metagenomics approaches, has revolutionized the study of the microbial ecology of foods. A better understanding of the resident microorganisms in food products can provide insights into the quality, shelf-life, and the potential risks and safety concerns. The survival of bacterial foodborne pathogens in a food is influenced not only by the chemical composition, physiological properties, and intrinsic and extrinsic factors, but also by the native microbial population. Many published studies have utilized metagenomics sequencing to analyze the microbial populations of fresh produce, cheeses, meats, fish, and fermented foods [1]. However, compared to other food categories, the microbiomes of low water activity foods (aw <0.80) are less defined but of interest due to the ability of some foodborne pathogens to persist in these foods for long periods of time. Understanding the types and relative populations of native microbiota in low water activity foods will provide quality and safety insights.

Only one published study has utilized high throughput metagenomics sequencing to examine the microbial population of a low water activity food: masala spice [2]. This study utilized targeted 16S rRNA sequencing to examine the microbiomes of different types of masala spices, all containing 8–17 individual spices including coriander, cumin, cinnamon, cloves, ginger, and nutmeg. It was determined that the masala spices had a high level of species diversity which varied by type (i.e., meat, chicken, garam) and the total number of combined spices, yet a core microbiota of *Proteobacteria*, *Firmicutes*, *Actinobacteria*, and *Bacteroidetes* could be resolved. This study has provided insights into microbial quality, spoilage and shelf-life potential, and the core resident bacterial taxa in masala spice. The microbiomes of other low water activity foods, including nuts, seeds, legumes, dried herbs, and other spices, have yet to be defined by high throughput sequencing technology.

Of current interest are the microbiomes of seeds and multi-commodity low water activity products made with seeds. Recent foodborne outbreaks and recalls in North America have been associated with pine nuts (the seeds of pines) [3–5], sesame seeds [6–8], and products made with these seeds [9–11]. In 2011, a multistate outbreak associated with *Salmonella enterica*-contamination of imported pine nuts in the U.S. resulted in 53 illnesses and two hospitalizations [5,12]. The implicated manufacturer recalled more than 21,000 pounds of pine nuts. In addition, three multistate salmonellosis outbreaks have occurred in the U.S. due to contaminated tahini (roasted sesame seed paste, often with oil). In 2011, consumption of contaminated tahini at three restaurants resulted in 23 illnesses with a 100% hospitalization rate [12,13]. In 2013, imported tahini was implicated in an outbreak which resulted in 17 illnesses, one hospitalization, and one death [12,14]. Recently, in 2019, imported tahini was also implicated in an outbreak which resulted in 6 illnesses and 1 hospitalization [11].

The aforementioned recalls and foodborne outbreaks associated with seeds highlight the importance of acquiring microbial population data on these food products. Since the microbiome of a food product can influence both its quality and safety, knowing the core microbiome, and observations of deviations from this baseline, can aid in food safety assessments. Therefore, the aim of this study was to elucidate the microbiomes of pine nuts and sesame seeds, including core microbiota.

## Materials and methods

### Seed samples used in this study

Ten different brands of hulled sesame seeds (*Sesamum indicum* L.) and hulled pine nuts (*Pinus pinea* L.) were acquired from the following vendors: Amazon (online distributor), Jewel (local retail grocer, Chicago, IL area), and Whole Foods (local retail grocer, Chicago, IL area). The growing and packaging/distribution locations of the different seeds are listed in S1 Table. Sesame seeds and pine nuts were stored in sealed original packaging at ambient for up to 1 mo prior to use.

### Total DNA extraction and 16S rRNA gene amplification

Triplicate 10-g samples of each sesame seed and pine nut brand were portioned into stomacher bags with 10 mL Butterfield’s Phosphate Buffer (BPB, pH 7.2; Thermofisher Scientific, Waltham, MA). Samples (n = 60) were homogenized for 1 min using a Seward 400 stomacher (Seward Laboratory Systems Inc., Davie, FL). Total DNA was extracted from 1 mL of each homogenate using the Qiagen DNeasy Blood and Tissue Kit (Qiagen Inc., Germantown, MD) according to the manufacturer’s instructions. DNA was quantified using the Qubit dsDNA BR Assay Kit (Invitrogen Carlsbad, CA). PCR was performed to amplify the V4 region of the16S rRNA genes from each sample as previously described [15].

### Library construction and sequencing

The 16s rRNA fragments were indexed using the Nextera XT Kit (Illumina, San Diego, CA) according to the manufacturer’s instructions as previously described [15]. The library, containing 60 samples, was diluted to 10 pM, spiked with 10% of 12.5 pM PhiX, and sequenced on a MiSeq with 600 cycles and V3 chemistry.

### 16S rRNA amplicon sequence analysis

Raw paired-end sequences were merged into consensus fragments and quality filtered as previously described [16]. The 16S rRNA gene sequences were taxonomically profiled to the genus-level using the *k*-mer-based classifier Kraken2 [17] and the Ribosomal Database Project 16S database [18]. Relative abundances were determined using Bracken 2.5 [19]. The relative abundances were averaged for the triplicate samples for each brand of pine nut and sesame seed.

### Metagenomic diversity analysis

The alpha diversity of the microbial population in the samples was estimated using Shannon, Simpson, inverse Simpson, and Chao1 indices using the vegan package 2.5–6 [20] in R 3.6.2. Significant differences between the indices for the pine nuts and sesame seeds were determined using Kruskal-Wallis non-parametric test. Principal coordinates analysis (PCoA) using the weighted UniFrac community distance metric [21] was used to compare the total diversity among different samples. Beta diversity was also evaluated using Bray-Curtis dissimilarity matrix; a multilevel pairwise comparison using Adonis (~Permanova) was calculated using package pairwiseAdonis [22]. A *p*-value less than 0.05 was considered significant.

## Results and discussion

### Microbial diversity in the pine nut and sesame seed microbiomes

**Fig 1:**
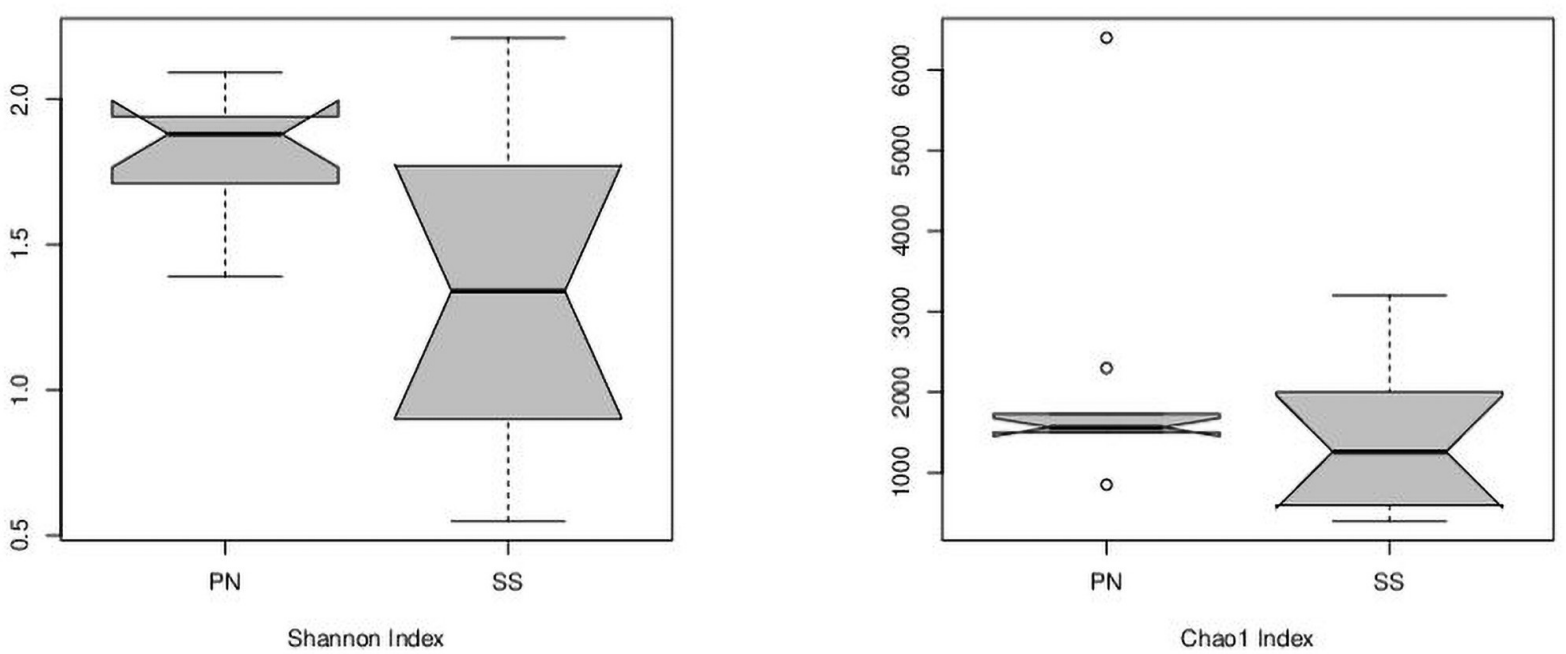
The alpha diversity of the microbial communities in sesame seed (SS; n = 10) and pine nut (PN; n = 10) microbiomes as measured by Shannon and Chao1 indices.

The alpha diversity of the pine nut and sesame seed samples were not significantly different as measured by Shannon and Chao1 indices, although there were differences observed between brands of the same seed type (Fig 1, S2 Table). The total unique taxa (OTU) identifications in the pine nut brands ranged from 57 to 141 with an average of 89, whereas identifications in the sesame seed brands ranged from 65 to 249 with an average of 128 (S2 Table). An overall correlation between seed growing locations and the number of taxa was determined. For pine nuts, the lowest number of taxa was observed in brands B (57), C (69), and G (61) for which the seed growing locations were Korea/Russia/Vietnam, Turkey, and Russia, respectively. The other brands had higher taxa identifications (81 to 141) and the seed growing locations were all in China. Brand A had the lowest diversity as measured by Shannon index, while brand F had the highest diversity. For sesame seeds, brands A (65), E (87), and H (69) had the lowest number of taxa identifications for which the seed growing locations were China, Mexico, and unlisted. The other brands with higher taxa identifications (97 to 249) had seed growing locations of India (with two brands having unlisted locations). Brand G had the lowest diversity, while brand E had the highest.

Approximately 90% of all pine nuts in the U.S. are imported from other countries, mainly China. Seven out of the 10 brands of pine nuts used in this study were imported from China, most likely accounting for the low microbial alpha diversity between the 10 different brands. Pine nuts grow in cones on pine trees, often high off the ground. The composition and nutrition contents of pine nuts harvested from different geographical locations can vary widely [23,24], suggesting that climate, soil type, and region-specific growing and harvesting practices play a role in pine nut composition. Similarly, the microbiome of pine nuts would also depend on these variables. Sesame seeds grow in pods on a plant closer to the ground. The more variable alpha diversity of the microbiome of sesame seeds may be due to the soil type, vicinity to wild animals and other crops, and to the diverse growing locations of the 10 different brands (i.e., China, India, Mexico, and unlisted).

**Fig 2:**
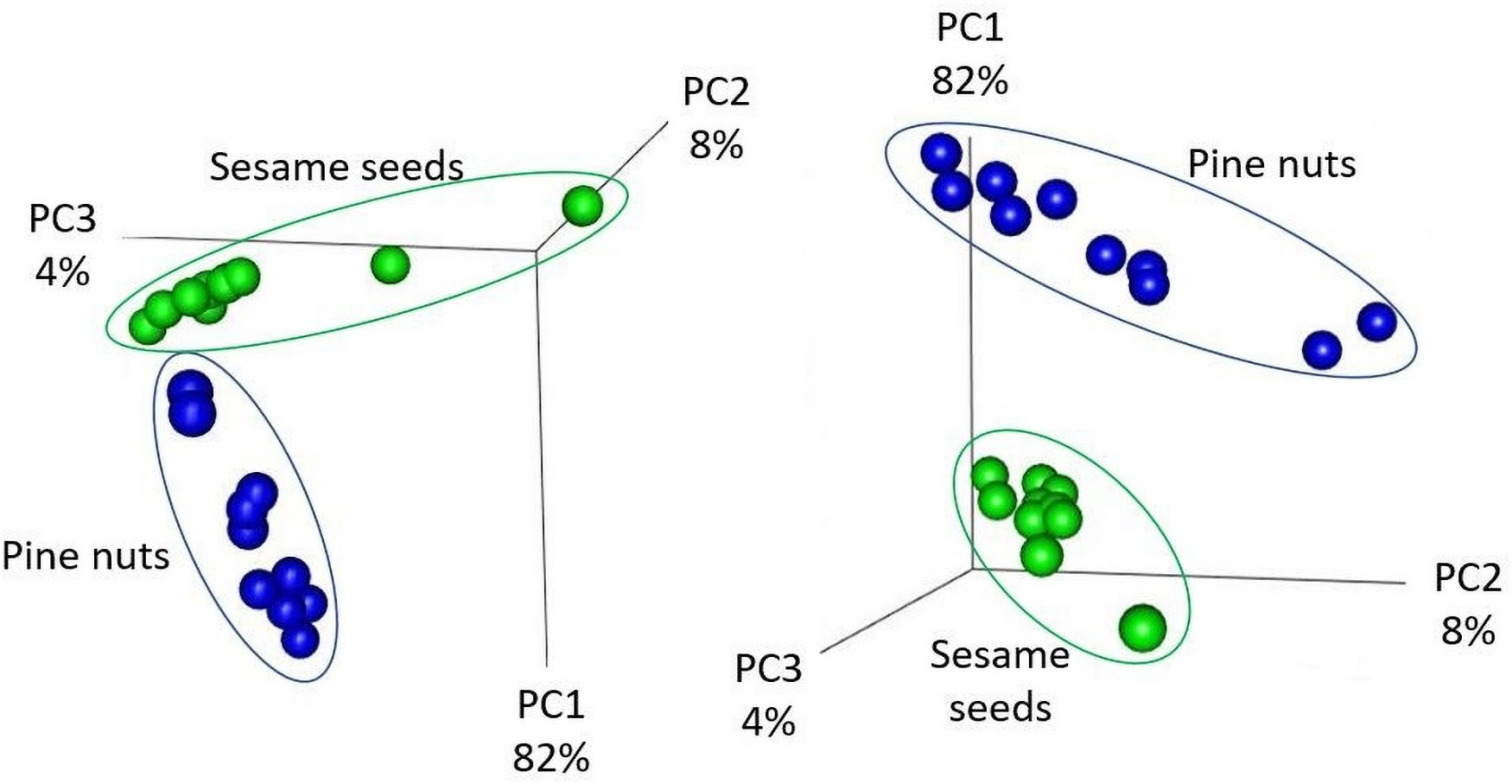
Principal coordinates analysis (PCoA) of the seed samples used in this study using the weighted UniFrac community distance metric. The 10 sesame seed brands (green) and pine nut brands (blue) cluster together.

Principal coordinates analysis (PCoA) was utilized to visualize the microbial beta diversity in the 10 pine nut brands and the 10 sesame seed brands. Results revealed that the same seed type clustered together (Fig 2). The first component represented 82% of the variation in beta diversity and was sufficient to separate the microbiome of the pine nuts from the sesame seeds. Interestingly, even though both pine nuts and sesame seeds are from the same food category (i.e., seeds), the microbiota of each are clearly separated. The second and third components, representing 8 and 4% of the beta diversity, separated the different brands within the same seed type. A pairwise comparison of the microbiomes between the different pine nut brands and between the different sesame seed brands revealed no significant differences (p = 1; data not shown).

### The pine nut microbiome

**Fig 3:**
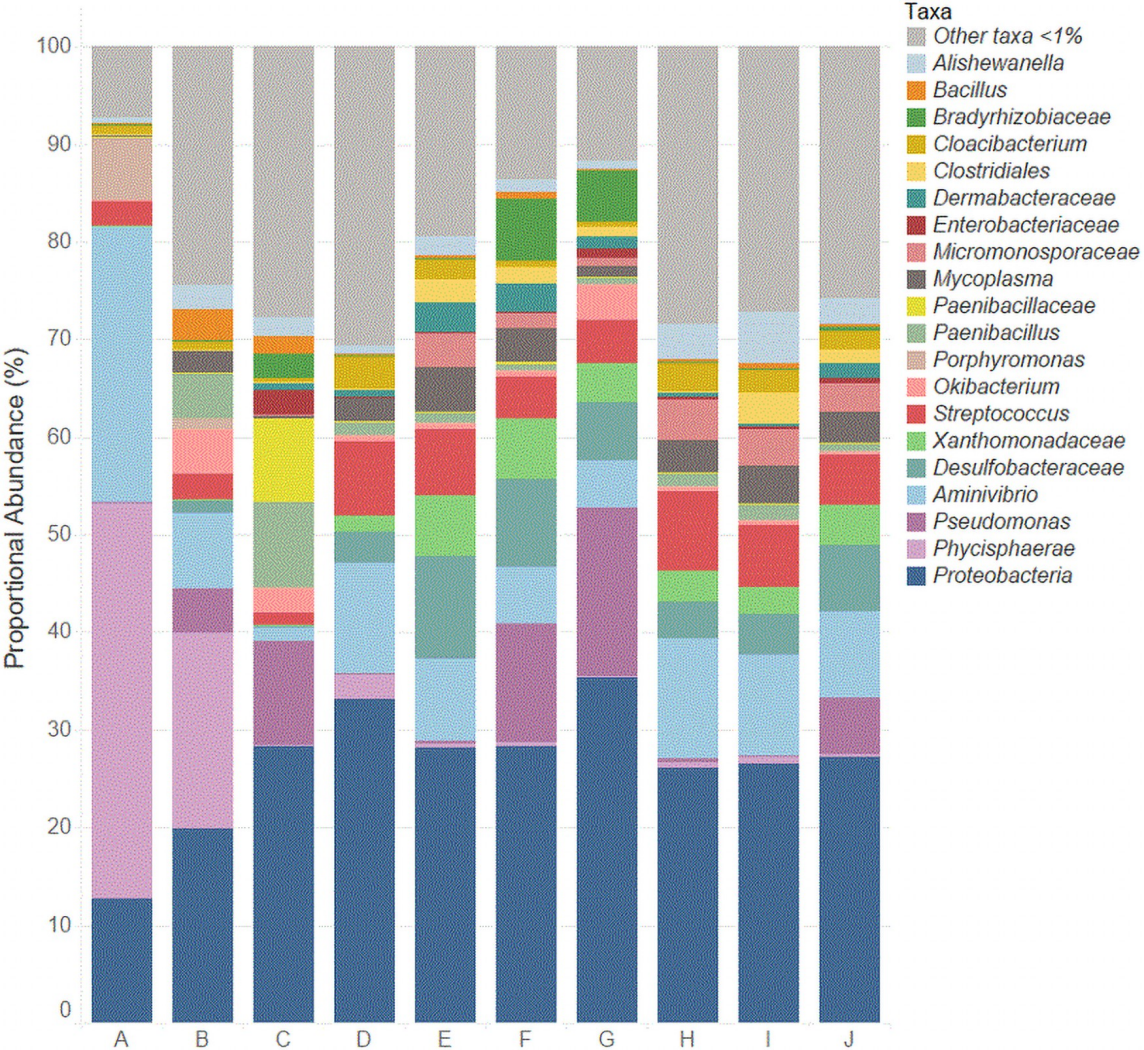
Relative abundance of unique taxa in the 10 brands of pine nuts.

In the 10 pine nut brands, 21 unique taxa were present with a proportional abundance >1% in at least one brand (Fig 3). The 21 taxa included 10 genera as well as unassigned OTUs in eight families, two classes (*Clostridiales* and *Phycisphaerae*), and one phylum (*Proteobacteria*). Nineteen of those taxa had proportional abundances >3% in at least one brand, and only 11 were >5% in at least one brand. The taxa with the largest proportional abundances included *Aminivibrio* (28.3% in brand A), *Phycisphaerae* (20.1 and 40.1% in brand A and B, respectively), *Pseudomonas* (17.3% in brand G) and *Proteobacteria* (35.4%, brand G). When comparing the microbiota of the 10 pine nut brands, of notable difference was the large abundance of *Phycisphaerae* in brands A and B, yet the abundance of this taxon in six of the other brands was <1% and it was not identified in brand C. Another notable difference included the large abundance of *Paenibacillus* (8.7%) and unassigned OTUs in the family *Paenibacillaceae* (8.5%) in brand C. This was also the only brand where pine nuts were cultivated in Turkey.

#### Core microbiome

The core microbiome of the pine nuts, defined as taxa with proportional abundances >1% and present in at least seven of the 10 brands, included *Alishewanella* (1.3–5.3%, 7 brands), *Aminivibrio* (1.5–28.3%, all 10 brands), *Mycoplasma* (1.0–4.6%, 8 brands), *Streptococcus* (1.2–6.8%, all 10 brands), unassigned genera in the families of *Desulfobacteraceae* (1.5–8.2%, 8 brands) and *Xanthomonadaceae* (1.7–6.2%, 7 brands), and unassigned OTUs in the phylum *Proteobacteria* (12.7–35.4%, all 10 brands). *Aminivibrio* and *Streptococcus* were the only genera present in all 10 brands with proportional abundances >1%.

#### Environmental microbiota

Of the 24 unique taxa identified in the pine nut brands with proportional abundance >1% in at least one brand, 14 of these taxa are considered ubiquitous environmental microbiota. Of the environmental taxa, the identified genera include *Alishewanella*, *Aminivibrio*, *Bacillus*, *Cloacibacterium*, *Okibacterium*, *Paenibacillus*, *Pseudomonas*, *and Streptococcus*. *Alishewanella*, a member of the core microbiome, was identified in all 10 pine nut brands at proportional abundances of 0.6–5.3%, with the greatest abundances observed in brands H and I. *Alishewanella* is a relatively new bacterial taxon and since its discovery in 1992 has been isolated from water and landfill soil [25,26]. *Alishewanella* is thought to play a role in the bioremediation process of chromate and sulfate [25] which provides insight into its existence in landfill and waste soil. *Alishewanella* has also been isolated from fermented foods [27] indicating its possible role in the fermentation process. This is the first occurrence where *Alishewanella* has been isolated in a seed product.

*Aminivibrio* was identified in all 10 pine nut brands at proportional abundances as low as 1.5% (brand C) to as high as 28.3% (brand A). *Aminivibrio* is a strictly anaerobic amino acid and organic acid-degrading microorganism which has been isolated from potato starch processing wastewater as well as the soil of rice fields [28,29]. Species of *Aminivibrio* are known to co-culture with hydrogen-utilizing methanogens [30] present in soil and waste of ruminants. As with *Alishewanella*, *Aminivibrio* was most likely in the soil where the pine nuts were cultivated.

Another ubiquitous environmental genus, *Bacillus*, was identified in all 10 pine nut brands, yet at very low abundances (<1%) in all brands except B and C where abundances were 3.1 and 1.8%, respectively. *Bacillus* species have been isolated recently from different soil types, including forest soil in China [31]. Since seven out of the 10 pine nut brands were cultivated in China, the finding of *Bacillus* is not unexpected. *Bacillus* species has also been identified in a wide variety of food products including dairy and powdered products [32,33], and powdered food products. *Bacillus* was also discovered to be a dominant microorganism in masala spice mixes, with proportional abundances as high as 13% [2]. Species in this genera are also known to be spoilage microorganisms as spores can survive heat treatment and thus lead to proliferation post-processing in many foods, including dairy products [34]. As such, identification of *Bacillus* in a food can provide information on its quality.

*Cloacibacterium* is a relatively new genus of bacteria found in diverse aquatic environments including sludge and wastewater [35,36]. In municipal wastewater and sludge, *Cloacibacterium* plays a role in breaking down the complex organic matter in these environments [37]. This genus was identified in all 10 pine nut brands with proportional abundances of 0.5–3.5%. the lowest abundance was determined in brand C (0.5%), where the pine nuts were cultivated in Turkey, and the highest abundances were in brands D, H, and I (3.5, 3.0, and 2.4%, respectively) where all pine nuts were cultivated in China.

*Okibacterium* is a relatively new plant-associated genus of bacteria containing two species. The species of *Okibacterium* have been isolated from plant roots and the seeds of plants [38,39]. Recently, *Okibacterium* was also isolated from ice wine during its fermentation process and was determined to be active based on endoenzymatic assays [40]. It is possible that *Okibacterium* was part of the microbiota of the berries used to make the ice wine. This genus was identified in all 10 pine nut brands with highest abundances in brands B (4.6%) and G (3.6%), both of which had the common growing location of Russia.

*Paenibacillus* was also identified in all 10 of the brands of pine nuts at proportional abundances ranging from 0.3–8.7%. *Paenibacillus* species are often isolated from soil and are associated with plant roots [41]. *Paenibacillus* has also been isolated from the rhizosphere of pine trees [42,43] and from pine litter [44]. Therefore, the finding of *Paenibacillus* in all ten of the pine nut brands is not surprising. This bacterial genus is commonly utilized for agricultural purposes. For example, certain *Paenibacillus* species are commonly used in biocontrol measures in agriculture [45] as well as a component in biofertilizers to promote plant growth [46]. Interestingly, *Paenibacillus* can inhibit the growth of the foodborne pathogen *Salmonella* during typical enrichment procedures [47], highlighting the need to optimize the enrichment protocols for pine nuts when assessing contamination with *Salmonella*.

Another ubiquitous environmental genera, *Pseudomonas*, was identified in all 10 pine nut brands at proportional abundances ranging from 0.8–17.3%. Large abundances of *Pseudomonas* were identified in brands C, F, and G (10.7, 12.2, and 17.3%, respectively). Since these three brands had different pine nut growing locations (i.e., Turkey, China, and Russia), *Pseudomonas* appears not to be location dependent and is part of the ubiquitous microbiota in the pine nut rhizosphere. Many of the species of *Pseudomonas* have been isolated from water and plants [48] and are beneficial to agriculture [49]. However, like *Bacillus*, *Pseudomonas* can also be an indicator of spoilage in foods, particularly in meats, dairy products, and vegetables [50–52]. Interestingly, also like *Bacillus*, *Pseudomonas* was identified at a high proportional abundance (19%) in Garam masala spice mixes [2].

*Streptococcus* is a prevalent microorganism found in a variety of environments including plants, soil, and different foods products [53] and species of this genus are also used in food fermentations [54]. *Streptococcus* was identified in all 10 pine nut brands ranging from 1.2% (brand C) to 8.2% (brand H). This bacterial genus has been previously identified in almonds [55]. *Streptococcus* is considered an indicator of soil contamination and also poor hygiene as it is present in fecal material [56]. As with *Pseudomonas*, *Streptococcus* appears not to be location dependent and is part of the ubiquitous microbiota in the pine nut rhizosphere.

#### Commensal microbiota

Commensals are microbes that are generally associated with host organisms and not often found in the environment. Commensal microbiota typically exist for the mutual benefit to the host; however, they can also cause disease and infection. In the pine nut brands, three commensal taxa were identified: *Mycoplasma*, *Porphyromonas*, and *Dermabacteraceae*. *Mycoplasma* was identified in all 10 of the pine nut brands in abundances ranging from 0.2% (brand A) to 4.6% (brand E). *Mycoplasma* are bacteria which lack a cell wall and survive by tightly adhering to hosts including humans, animals, plants, and insects [57]. This taxon is widespread in the environment and can be saprophytic or parasitic in nature. In immune compromised humans, certain species of *Mycoplasma* can invade the respiratory tract and cause diseases including pneumonia [57]. On plants, *Mycoplasma*-associated disease can be economically costly. Due to its association with plants and animals, the finding of *Mycoplasma* in all of the pine nut brands is not unanticipated.

*Porphyromonas* is a bacterial taxon identified in 9 out of 10 pine nut brands ranging from <0.1% (brands C, E, G, and J) to 6.3% (brand A); the taxon was not identified in brand F. Species of *Porphyromonas* are commensal and found in the oral microbiome of animals, most notably humans [58]. Although this taxon is present in healthy individuals, certain species of *Porphyromonas* can cause human periodontal disease [59]. In addition, *Porphyromonas* is present in human and animal fecal material [60], possibly explaining its identification in the pine nut brands in this study.

The bacterial family *Dermabacteraceae* includes the commensal genera *Brachybacterium*, *Dermabacter*, *Devriesea*, and *Helcobacillus*. Unclassified OTUs of *Dermabacteraceae* were identified in 8 of the 10 pine nut brands; it was not identified in brands A and C. the highest abundances were identified in brands E and F (3.1 and 3.0%, respectively), where both brands of pine nuts were cultivated in China. Species in this taxon have been isolated from both the environment [61] and human wounds [62]. Interestingly, *Brachybacterium*, a genus within *Dermabacteraceae*, has also been isolated from the surfaces of cheeses [63] from fermented seafood [64].

#### Taxa containing potential human foodborne pathogens

Taxa identified in the pine nut brands containing potential human foodborne pathogens include the genus *Bacillus* and the family *Enterobacteriaceae*. As mentioned previously, *Bacillus* was identified in all 10 pine nut brands at abundances of <1–3.1%. In addition to its ubiquitous environmental presence, *Bacillus* also contains species of human foodborne pathogens, most notably *Bacillus cereus* [65]. Unclassified OTUs of *Enterobacteriaceae* were identified in 6 out of 10 pine nut brands at abundances of <1%, with the exception of brand C with the unique growing location of Turkey, where the abundance was 2.5%. The family *Enterobacteriaceae* contains many genera of Gram-negative bacteria, some of which can be associated with foodborne illness including *Escherichia*, *Klebsiella*, *Salmonella*, and *Shigella* [66]. *Enterobacteriaceae* have been identified as part of the microbiomes of a variety of other food products including spice mixes, fresh produce, and dairy products [2,67–69]. In addition, the presence of *Enterobacteriaceae* in a food matrix is known to hinder the enrichment of the foodborne pathogen *Listeria monocytogenes* [70], highlighting the need for efficient enrichment procedures when *Enterobacteriaceae* are present in foods.

#### Notable between-brand differences

While the relative abundances of each taxa fluctuated between the pine nut brands, the most striking difference in the microbiota observed between brands was in high relative abundance of unclassified OTUs of the class *Phycisphaerae* in brands A and B (40.6 and 20.1%, respectively). The taxon *Phycisphaerae* includes water-dwelling bacteria which have been identified from aqueous environments [71,72]. The finding of *Phycisphaerae* in brands A and B most likely indicates that the water used for cultivation of the pine nuts contains that class of bacteria. Interestingly, *Phycisphaerae* was not identified in brand C, which was the only brand in which the pine nuts were cultivated outside of Asia.

### The sesame seed microbiome

**Fig 4:**
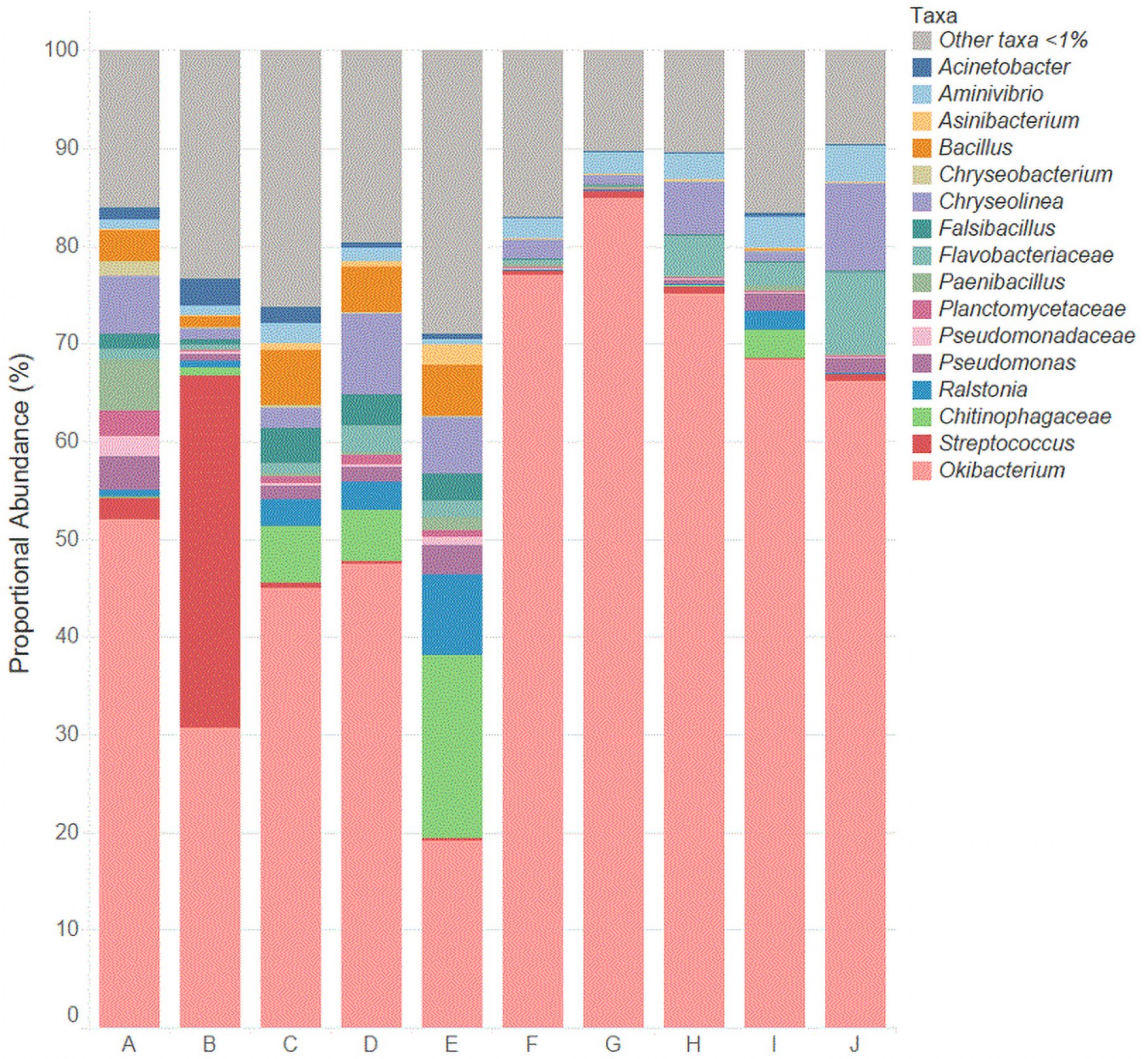
Relative abundance of unique taxa in the 10 brands of sesame seeds.

In the 10 sesame seed brands, 16 unique taxa were present with a proportional abundance >1% in at least one brand (Fig 4). These 16 taxa included 12 genera as well as unassigned OTUs in three families (*Chitinophagaceae*, *Flavobacteriaceae*, and *Pseudomonadaceae*) of one phylum (*Plantomycetes*). Out of the 16 taxa, eight had proportional abundances >5% in at least one brand and only three (*Okibacterium*, *Streptococcus*, and *Chitinophagaceae*) had abundances >10% in at least one brand. *Okibacterium* was the only taxon present at >1% in all 10 brands of sesame seeds and ranged from 19.2% (brand E) to 84.9% (brand G). Other taxa with high proportional abundances included *Streptococcus* (36.0% in brand B) and *Chitinophagaceae* (18.8% in brand E). When comparing the microbiota of the 10 different brands of sesame seeds, it was observed 13 taxa had proportional abundances >1% in brand A, however only 2 taxa (*Okibacterium* and *Aminivibrio*) were >1% in brand G.

#### Core microbiome

The core microbiome of the sesame seeds, defined as taxa with proportional abundances >1% and present in at least seven of the 10 brands, included *Aminivibrio* (1.0–3.7%, 8 brands), *Chryseolina* (1.0–9.1%, 7 brands), *Flavobacteriaceae* (1.1–8.6%, 7 brands), and *Okibacterium* (19.2–84.9%, all 10 brands). *Okibacterium* was the only member of the core microbiome identified in all 10 sesame seed brands.

#### Environmental microbiota

All of the 16 unique taxa with proportional abundances >1% in at least one brand which were identified in the sesame seeds are considered ubiquitous environmental microbiota. The genus level identifications include *Acinetobacter*, *Aminivibrio*, *Asinibacterium*, *Bacillus*, *Chryseobacterium*, *Chryseolina*, *Falsibacillus*, *Okibacterium*, *Paenibacillus*, *Pseudomonas*, *Ralstonia*, and *Streptococcus*. The genera *Aminivibrio*, *Bacillus*, *Okibacterium*, *Paenibacillus*, *Pseudomonas*, and *Streptococcus* were also identified as members of the pine nut microbiome with proportional abundances >1% in at least one of the 10 brands. Sesame seeds had much higher abundances of *Okibacterium* (19.2–84.9%) compared to pine nuts (0.2–4.6%). In addition, *Streptococcus* abundances varied widely in the sesame seeds (<1–36.0%) compared to the more uniform distribution in pine nuts (1.2–8.2%).

*Acinetobacter*, a ubiquitous environmental genus, was identified in the sesame seed brands at proportional abundances ranging from 0.1% (brands F, G, and J) to 2.8% (brand B). Species of *Acinetobacter* have been isolated from soil and water [73,74] and are often the focus of biotechnology research due to their ability to degrade certain pollutants. *Acinetobacter* can proliferate in many diverse environments and can become the predominate taxon under well-aerated conditions [75]. Therefore, *Acinetobacter* was most likely present in the soil where the sesame seeds were cultivated.

Another environmental genus, *Asinibacterium*, was identified at a proportional abundance of 2.2% in brand E, yet not identified in brands A, G, or H. Brand E was the only brand where sesame seeds were cultivated in Mexico. However, brands A, G, and H all had a different cultivation location and therefore no correlation between cultivation location and *Asinibacterium* identification could not be determined. *Asinibacterium* has been isolated from heavy metal-contaminated subsurface sediments [76] and can thus potentially be used for bioremediation purposes. In addition, *Asinibacterium* has also been isolated from one other low a_w_ food product: donkey milk powder [77].

*Chryseolina* is an environmental taxon that has been repeatedly isolated from the soil and is considered a part of the soil microbiome [78]. This genus was identified in all of the sesame seed brands in proportional abundances ranging from <1% (brand G) to 9.1% (brand J). Although not much is known about this genus, *Chryseolina* was most likely present in the soil where the sesame seeds were cultivated.

The environmental bacterial genus, *Falsibacillus*, was identified in four sesame seed brands at abundances >1% (brands A, C, D, and E). *Falsibacillus* are considered rhizobacteria and have only ever been isolated from soil and plants [79]. The presence of this taxon in some of the sesame seed brands is therefore not surprising.

#### Commensal microbiota

The genera *Chryseobacterium* and *Ralstonia*, in addition to their ubiquitous environmental presence, are also considered commensal microorganisms. *Chryseobacterium* has been identified in plants and soil [80]. Species of this genus have also been identified as commensal organisms of insects including moths and mosquitoes [81]. *Chryseobacterium* was identified in sesame seed brand A at an abundance of 1.4%; however, this taxon was identified at <1% in all other brands. Similar to *Asinibacterium*, it appears that this taxon was most likely present in the soil where the sesame seeds of brand A were cultivated.

*Ralstonia* is a genus of bacteria which has been identified in soil, water, and plants [82] and are also commensals of humans [83]. Certain species of *Ralstonia* are members of the human oral cavity and the upper respiratory tract of healthy individuals [83]. *Ralstonia* can revert from a commensal organism to a pathogen in people who are immune compromised. Species of *Ralstonia* are also devastating plant pathogens and cause lethal wilting disease in over 200 plant species [84]. Although *Ralstonia* was identified in all 10 sesame seeds brands, this taxon was at abundances >1% in only four brands (C, D, E, and I). Brand E sesame seeds had the highest proportional abundance of *Ralstonia* (8.3%) and was also the only brand where the sesame seeds were cultivated in Mexico.

#### Taxa containing potential human foodborne pathogens

Similar to the pine nut brands, *Bacillus* was also identified in the sesame seed brands with proportional abundances ranging from <1% (brands F, G, H, I, and J) to 5.8% (brand C). In the sesame seed brands, *Bacillus* was the only taxon identified which contains human foodborne pathogens. Although this genus contains species which can cause foodborne illness, *Bacillus* is also a ubiquitous environmental pathogen and has been isolated from soil [85] and a variety of food products [32,33].

#### Notable between-brand differences

The total number of unique taxa in each of the brands of sesame seeds ranged from two (brand G) to 13 (brand A). In brand G, only *Okibacterium* and *Aminivibrio* were identified with proportional abundances >1%; this brand was one of four brands in which the sesame seeds were cultivated in India. Interestingly, brand A was the only brand in which the sesame seeds were cultivated in China (although some of the brands had unlisted growing and harvesting locations), indicating the difference in the soil microbiome in this location. Another notable difference is the high abundance of *Streptococcus* in brand B (36.0%) compared to the abundance in all other brands (0.2–2.2%). As mentioned previously, *Streptococcus* is considered an indicator of soil contamination as this taxon is present in fecal material [56].

### Conclusions

The microbiomes associated with different food products are important in that they define the overall safety and quality. Metagenomics research is becoming more prevalent in food safety research, however there is a dearth of information related to low water activity foods. This study examined the microbiomes of ten different brands of pine nuts and sesame seeds and defined the associated core microbiomes and types of microbiota present. It is noted that this study did not examine species-level identifications and therefore no foodborne pathogens were identified in this study. Future studies could further explore the bacterial species associated with seeds. Overall, the results from this study provide insights into food safety issues including presence of spoilage organisms which may affect shelf life potential and identification of bacterial genera which may co-enrich with foodborne pathogens, possibly hindering detection.

## Supporting information

S1 Table Growing packaging/distribution locations of the ten different brands of pine nuts and sesame seeds assessed in this study. (XLSX)

S2 Table The unique taxa identifications and alpha diversity metrics of the ten different brands of pine nuts and sesame seeds. (XLSX)
